# Immunoglobulin A nephropathy with massive paramesangial deposits caused by anti-vascular endothelial growth factor therapy for metastatic rectal cancer: a case report and review of the literature

**DOI:** 10.1186/1756-0500-6-450

**Published:** 2013-11-09

**Authors:** Mayumi Yahata, Izaya Nakaya, Tsutomu Sakuma, Hiroshi Sato, Shigehisa Aoki, Jun Soma

**Affiliations:** 1 Division of Nephrology, Iwate Prefectural Central Hospital, 1-4-1 Ueda, Morioka, Iwate 020-0066, Japan; 2 Division of Pathology, Iwate Prefectural Central Hospital, 1-4-1 Ueda, Morioka, Iwate 020-0066, Japan; 3 Department of Clinical Pharmacology and Therapeutics, Tohoku University Graduate School of Pharmaceutical Sciences and Medicine, Tohoku University, Sendai 980-8578, Japan; 4 Department of Pathology & Biodefense, Faculty of Medicine, Saga University, Saga 849-8501, Japan

**Keywords:** Bevacizumab, Nephrotic syndrome, Anti-vascular endothelial growth factor therapy, Immunoglobulin A nephropathy

## Abstract

**Background:**

Bevacizumab, a recombinant humanized monoclonal antibody for vascular endothelial growth factor, has been widely used in various cancers offering substantial clinical benefit. It is reportedly associated with development of high-grade proteinuria and nephrotic syndrome with the histology of thrombotic microangiopathy, but there has been no report describing the development of immunoglobulin A nephropathy in bevacizumab-treated patients.

**Case presentation:**

A 68-year-old man with metastatic rectal cancer was treated with bevacizumab. He presented with hematuria and proteinuria 15 and 17 months, respectively, after bevacizumab initiation. Bevacizumab was stopped at 17 months. Renal biopsy at 19 months revealed immunoglobulin A nephropathy, with numerous paramesangial hemispherical deposits and thrombotic microangiopathy. Electron microscopy showed numerous paramesangial electron-dense deposits of various sizes, and subendothelial injuries. Proteinuria almost completely resolved 8 months after bevacizumab cessation, although hematuria persisted. Follow-up renal biopsy 11 months after bevacizumab cessation showed a marked decrease in mesangial immunoglobulin A deposits and paramesangial electron-dense deposits, which correlated with a gradual decrease in serum immunoglobulin A.

**Conclusion:**

This is the first case report that confirmed histologically the development and resolution of immunoglobulin A nephropathy during and after bevacizumab therapy. This case shows that there may be other mechanisms of glomerular injury by bevacizumab besides glomerular endothelial injury leading to thrombotic microangiopathy.

## Background

Vascular endothelial growth factor (VEGF) is an endogenous glycoprotein that plays a critical role in the growth of blood vessels [1]. Bevacizumab, a recombinant humanized monoclonal antibody for VEGF, inhibits tumor angiogenesis, and the addition of bevacizumab to chemotherapy is effective in the treatment of advanced or metastatic cancers, including breast cancer, colorectal cancer, and non-small cell lung cancer, renal cell carcinoma, and glioblastoma multiforme [2]. However, the addition of bevacizumab to chemotherapy increases the risk of high-grade proteinuria and nephrotic syndrome [3]. Eremina *et al.*[4] reported that thrombotic microangiopathy (TMA) was common in patients treated with bevacizumab and that, in human renal biopsy specimens and an animal model, a decrease in glomerular VEGF induced direct damage of the glomerular endothelium.

We describe the case of a patient with metastatic rectal cancer treated with bevacizumab, who developed nephrotic syndrome with the histology of immunoglobulin (Ig) A nephropathy with massive paramesangial deposits, along with mild TMA. Proteinuria resolved following bevacizumab cessation, and marked decreases in both IgA and paramesangial electron-dense deposits (EDDs) were confirmed by follow-up biopsy 11 months after bevacizumab cessation.

## Case presentation

A 68-year-old man with no remarkable medical history underwent rectal low anterior resection in November 2008 for Stage T3N0M0 rectal cancer. In December 2009, pulmonary metastasis was found and capecitabine, oxaliplatin, and bevacizumab treatment (400 mg every 3 weeks) initiated. At chemotherapy initiation, his serum creatinine was 0.66 mg/dl. Urinalysis was done every month after the initiation of chemotherapy, but neither proteinuria nor hematuria was evident. In March 2011 (15 months after chemotherapy initiation), dipstick urine evaluation first demonstrated hematuria of 1–2+. Proteinuria was evident for 2 months afterward. Subsequently, chemotherapy was stopped at the end of May 2011. However, heavy proteinuria persisted, and the patient was referred to us in July 2011. He had gained 6 kg of body weight and had developed edema of the lower extremities over the preceding month. His blood pressure was 135/80 mm Hg. Laboratory investigations are listed in Table 1. Urinalysis showed proteinuria of 3.5 g/day and >100 red blood cells/high-power field (HPF), with granular and fatty casts. Marked hypoproteinemia and hypoalbuminemia were observed, but serum creatinine was normal. Serum IgA was elevated to 487 mg/dl (normal: 110–410 mg/dl). Serum complement component (C) 3 and C4, total serum hemolytic activity, and hepatobiliary function were normal. Tests for hepatitis B and C, antinuclear antibody, anti–double-stranded deoxyribonucleic acid antibody, and cryoglobulins were all negative. Carcinoembryonic antigen (CEA) was elevated to 11.5 ng/ml (normal: <5 ng/ml), but carbohydrate antigen 19–9 was within normal range. No red blood cell fragmentation was observed. Computed tomography showed normal-sized kidneys, massive ascites, and bilateral pleural effusion, and a small, isolated pulmonary metastatic lesion was observed in the right inferior lobe.

**Table 1:** Laboratory data at the time of the first biopsy

Urinalysis		Triglycerides	154 mg/dll
Protein	3.5 g/day	Asparate aminotransferase	40 IU/l
Glucose	(−)	Alkaline phosphatase	32 IU/l
Urinary red blood cells	> 100/HPF	Lactate hedydrogenase	230 IU/l
Granular casts	(+)		
Fatty casts	(+)	Serology	
		Immunoglobulin G	571 mg/dl
Peripheral blood		Immunoglobulin A	487 mg/dl
White blood cells	3.17 × 10^3^/μl	Immunoglobulin M	66 mg/dl
Red blood cells	3.78 × 10^6^/μl	Total serum hemolytic activity	41.8 U/ml
Hemoglobin	13.0 g/dl	Complement component 3	81 mg/dl
Hematocrit	38%	Complement component 4	20 mg/dl
Platelets	64 x 10^3^/μl	Cryoglobulin	(−)
Red blood cell fragmentation	(−)	Antinuclear antibody	(−)
		Anti-dsDNA antibody	(−)
Biochemistry		Hepatitis B virus antigen	(−)
Urea nitrogen	9.4 mg/dl	Hepatitis C virus antibody	(−)
Serum creatinine	0.65 mg/dl		
Uric acid	6.0 mg/dl	Tumor marker	
Total protein	4.8 g/dl	Carcinoembryonic antigen	11.5 ng/ml
Serum albumin	2.2 g/dl	Carbohydrate antigen 19-9	< 2.0 U/ml
Total choresterol	198 mg/dl		

Foot notes of Table 1.

dsDNA, double-stranded deoxyribonucleic acid antibody.

**Figure 1:**
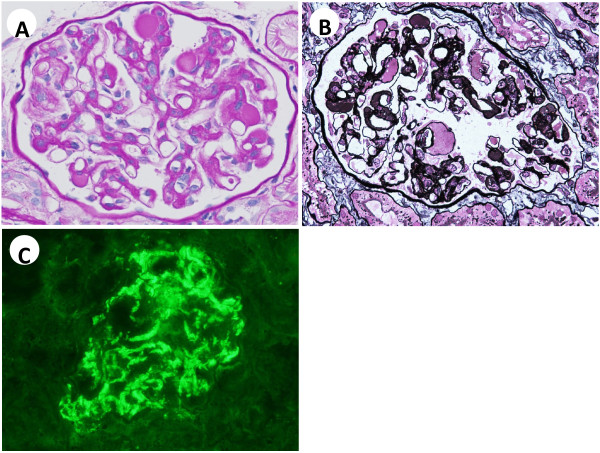
**Light microscopy findings at the first biopsy. (A)** Many periodic acid-Schiff (PAS) positive paramesangial hemispherical deposits were observed, and hyaline-like materials occupied dilated capillary lumens. At the nine o’clock position, mesangiolysis was also observed. **(B)** Subendothelial areas were markedly widened, and filled with hyaline-like materials, which were stained weaker than paramesangial hemispherical deposits. **(C)** Strong IgA deposition was observed in the mesangium and along capillary walls. **(A)** PAS staining; original magnification 400×. **(B)** Periodic acid-methenamine-silver staining; original magnification 400×.

**Figure 2:**
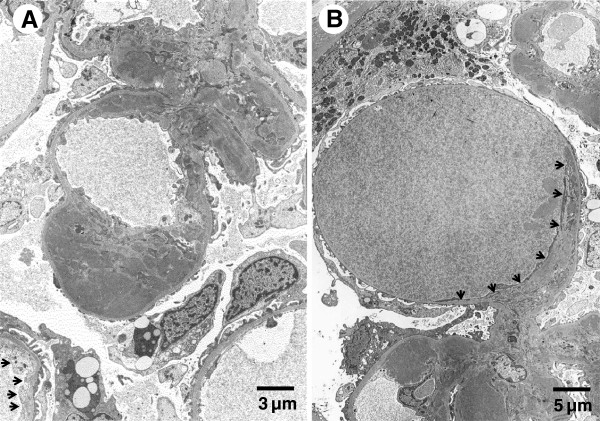
**Electron microscopy findings at the first biopsy. (A)** Numerous paramesangial electron-dense deposits (EDDs) of various sizes and segmental widening of subendothelial areas with electron-lucent materials were evident (arrows). **(B)** Giant globular materials widened the subendothelial space remarkably and compressed endothelial cells to the opposite side (arrows). Paramesangial and subendothelial EDDs were also seen in the lower and upper parts of the photograph, respectively.

Percutaneous renal biopsy was performed. Three out of forty-three glomeruli were globally sclerotic. Other glomeruli exhibited mild-to-moderate mesangial proliferation, with periodic acid-Schiff (PAS)-positive paramesangial hemispherical deposits. Capillary lumens were dilated and occupied by PAS-positive material. Mesangiolysis was also observed in some glomeruli (Figures 1A and B). Tubular atrophic and interstitial fibrotic changes were observed focally. Arterial vessels showed mild sclerosis, with no intra-arterial or intra-arteriolar thrombi. Immunofluorescence (IF) study revealed marked intensity (+++) for IgA (Figure 1C) and C3, and slight intensity (+) for IgG, IgM, C1q, and fibrinogen, which were found mainly in the mesangium and along capillary walls. Electron microscopy revealed numerous EDDs in paramesangial areas, which were fine granular, spherical, or hemispherical in shape and varying in size (Figure 2A). Similar EDDs were observed in some subendothelial areas. In addition, subendothelial electron-lucent widening with loose granular materials was observed focally and segmentally. Some glomerular capillary lumens were occupied with globular and loose materials, which were thought to be serum components. These materials gathered in subendothelial areas, compressing endothelial cells to the opposite side (Figure 2B).

The patient underwent follow-up for nephrotic syndrome with unremarkable therapy. Heavy proteinuria with hematuria continued after bevacizumab cessation, but began to decrease gradually after 6 months. Eight months later, proteinuria had decreased to 0.3 g/g · creatinine, and serum total protein and albumin were 7 g/dl and 4 g/dl, respectively. However, hematuria persisted.

**Figure 3:**
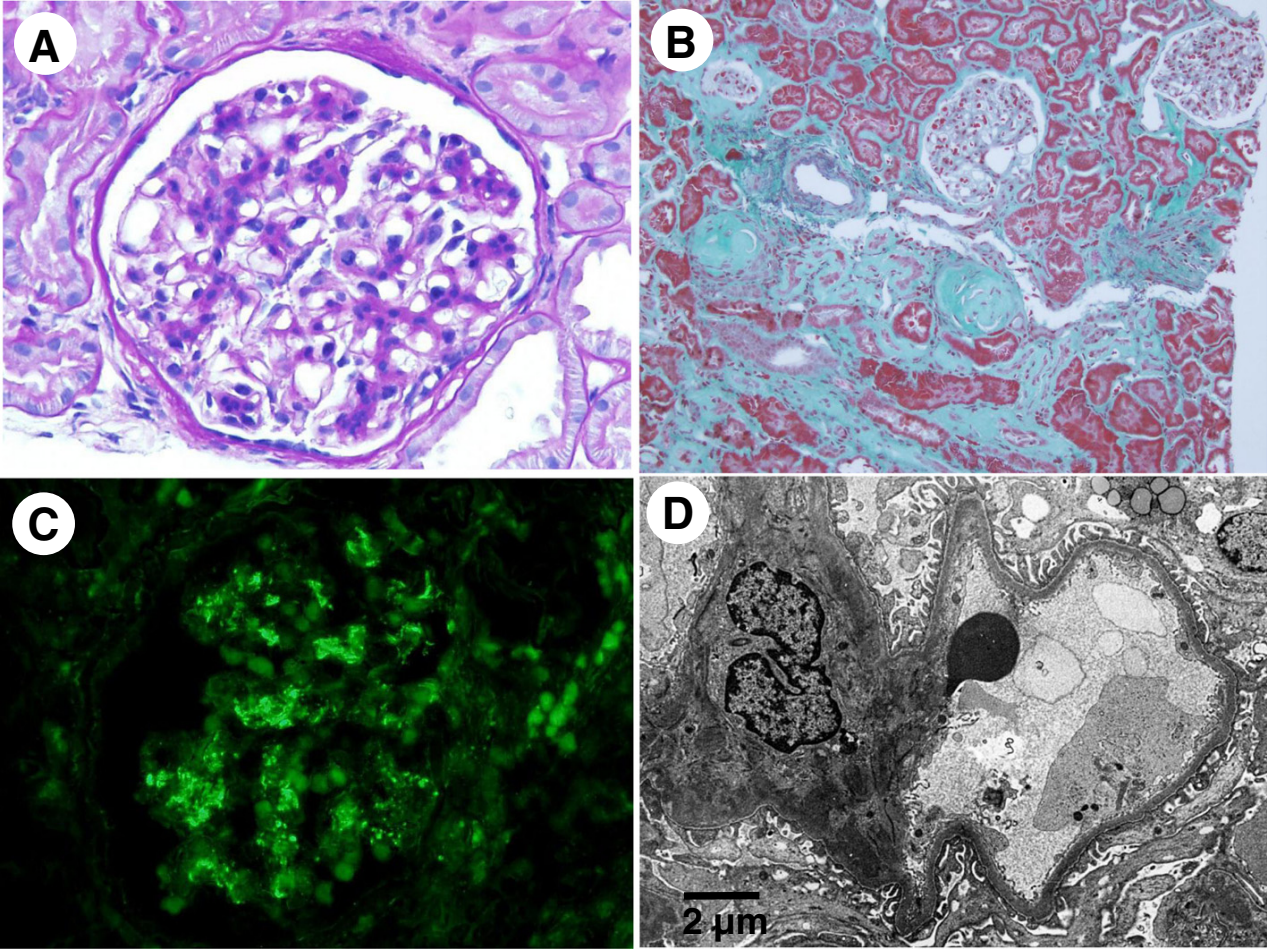
**Light and electron microscopy findings at the second biopsy. (A)** Mild mesangial proliferation was seen, but neither definite paramesangial hemispherical deposits nor widened subendothelial spaces were evident. **(B)** Two glomeruli showed mild mesangial proliferation. However, two glomeruli were globally sclerotic and one had collapsed (ten o’clock position); in addition, tubular atrophy and interstitial fibrosis was evident around these glomeruli. **(C)** The degree of IgA deposition had clearly decreased compared with the first biopsy. **(D)** Electron-dense deposits in the mesangium and subendothelial area were obviously decreased compared with the first biopsy. **(A)** Periodic acid-Schiff (PAS) staining; original magnification 300×. **(B)** Elastica Masson staining; original magnification 100×.

In June 2012, a second renal biopsy was performed to reevaluate renal histology. At the second biopsy, serum creatinine was 1.09 mg/dl with negative urinary protein and urinary red blood cells of 20–29/HPF (Table 2). Total protein and serum albumin were normalized, and serum IgA had decreased from 487 mg/dl at the first biopsy to 297 mg/dl. Four out of twenty-nine glomeruli were globally sclerotic. Other glomeruli exhibited mild mesangial proliferation only (Figure 3A). Tubular atrophy and interstitial fibrosis were present in approximately 30% of the tubulointerstitial area (Figure 3B). IF revealed mild intensity (+) for IgA (Figure 3C) and C3, decreased compared with the first biopsy. On electron microscopy, EDDs in the mesangium were decreased in number and size compared with the first biopsy (Figure 3D). Widening of subendothelial spaces and subendothelial EDDs were still observed segmentally (data not shown).

**Table 2:** Laboratory data at the time of the second biopsy

Urinalysis	
Protein	(−)
Glucose	(−)
Urinary red blood cells	20-29/HPF
Casts	(−)
Biochemistry	
Urea nitrogen	21.6 mg/dl
Serum creatinine	1.09 mg/dl
Uric acid	7.5 mg/dl
Total protein	6.9 g/dl
Serum albumin	4.5 g/dl
Serology	
Immunoglobulin G	928 mg/dl
Immunoglobulin A	297 mg/dl
Immunoglobulin M	57 mg/dl

The pulmonary metastatic lesion showed only slight enlargement, and no new metastatic lesions were identified. However, capecitabine treatment (1800 mg/day) was initiated at the end of October 2012 because CEA gradually increased to 11.4 ng/ml. In December 2012, laboratory data showed no proteinuria, urinary blood cells of 10–15/HPF, serum creatinine of 0.82 mg/dl, and serum IgA of 280 mg/dl. At the final follow-up in July 2013, hematuria first disappeared, and serum creatinine and serum IgA were 0.84 mg/dl and 270 mg/dl, respectively.

## Conclusions

Our patient was diagnosed with bevacizumab-induced IgA nephropathy with massive paramesangial deposits because of the following:

•neither proteinuria nor hematuria were identified by medical checkups performed prior to bevacizumab treatment

•urinary abnormalities first appeared 14 months after bevacizumab initiation

•proteinuria disappeared, and hematuria decreased, after bevacizumab cessation and

•IgA deposition and paramesangial EDDs were markedly decreased at the second biopsy, 10 months after bevacizumab cessation

TMA, characterized by subendothelial widening with loose granular materials, was also evident in our patient.

Bevacizumab, a recombinant humanized monoclonal antibody for VEGF, inhibits VEGF-induced angiogenesis and tumor growth [5]. Anti-VEGF agents are generally well tolerated, but hypertension and asymptomatic proteinuria are common dose-related adverse effects, frequently occurring together [6]. The incidence of proteinuria ranges from 21%–63% [6]. Although this proteinuria is largely asymptomatic and low-grade, nephrotic-range proteinuria occurs in 1%–2% of bevacizumab-treated patients [7]. VEGF is constitutively expressed on podocytes, and its receptors exist on glomerular capillary endothelial cells [8]. Maintenance of the structure and function of the glomerulus necessitates continuous VEGF production by podocytes. VEGF decrease causes the injury of fenestration of the endothelium, promoting the development of endothelial injuries [4]. These findings suggest that VEGF decrease compromises the glomerular capillary permeability barrier.

Table 3 shows biopsy-documented glomerular disease in 15 bevacizumab-treated patients, including our case [4,5,9-14]. The period from bevacizumab initiation to glomerular disease onset ranged from 2 weeks to 15 months. Eight patients exhibited nephrotic syndrome. Nine patients were followed-up after bevacizumab cessation, and all except one experienced decrease or resolution of proteinuria. Histologically, TMA was evident in 12 patients: two in whom it was superimposed on collapsing glomerulopathy or mesangial proliferative glomerulonephritis (GN; our case), one with collapsing glomerulopathy alone, one with cryoglobulinemic GN, and one with focal proliferative immune complex GN (IgM-type). Of the 12 patients with TMA, predominant glomerular IgA deposition was observed in five, including our case [4,9,13]. In two of these cases, EDDs were absent. IgA deposition in these patients could be nonspecific; for example, it could be caused by IgA trapped by damaged capillary walls [12]. In one patient with renal cell carcinoma [9], IgA-dominant glomerular deposition was found at renal biopsy, although no IgA deposition was evident in tissue samples obtained during nephrectomy before bevacizumab treatment. However, EDDs were mainly distributed in widened subendothelial areas associated with mesangial cell interposition. These histological features and the absence of hematuria, which were inconsistent with IgA nephropathy, led to a diagnosis of IgA-predominant immune complex GN rather than IgA nephropathy [9]. In another patient [4], EDDs were observed mainly in subendothelial areas and segmentally in the mesangium. In our case, the first symptom was hematuria, which was followed by proteinuria. Histologically, IF revealed IgA deposition mainly in the mesangium, along with EDDs and numerous paramesangial hemispherical deposits, which are characteristic of IgA nephropathy [15]. After bevacizumab cessation, these deposits markedly decreased, with the resolution of proteinuria and decreased hematuria. Interestingly, sorafenib, another human monoclonal antibody for VEGF, was reported to cause a rapid deterioration of renal function and a marked increase of proteinuria in pre-exising IgA nephropathy [16]. Chronological changes of our and the reported cases emphasize the close relationship between anti-VEGF effect and IgA nephropathy development and progression. Notably, glomerular and tubulointerstitial sclerotic changes evident at the first biopsy had clearly progressed at the second, associated with increased serum creatinine (0.66 mg/dl at the first biopsy and 1.09 mg/dl at the second), despite the fact that proteinuria had resolved and hematuria had decreased at the second biopsy. This may be because IgA nephropathy remained “active” during the first 6 months after bevacizumab cessation. No decrease in proteinuria and hematuria during the first 6 months supports this possibility.

**Table 3:** Biopsy-documented cases with glomerular disease by bevacizumab

**Reference**	**Age (years)/gender**	**Onset**	**Proteinuria**	**Hematuria**	**LM**	**IF**	**EM**	**Follow-up**
4	59/M	9 mo	3.4 g/gCr	ND	Glomerular TMA	ND	FPE	Proteinuria decreased
4	74/M	3 mo	2.7 g/gCr	ND	Glomerular TMA	ND	FPE	Proteinuria decreased
4	56/M	7 mo	0.16 g/day	ND	Glomerular TMA	ND	Focal FPE	Dead shortly thereafter
4	62/M	3 mo	0.5 g/dl	+	Glomerular TMA	IgA, MES	Fibrin, SEn and MES	Proteinuria resolved
4	61/M	5 mo	4.6 g/day	ND	Glomerular TMA	ND	Focal podocyte injuries	ND
4	59/F	9 mo	0.8 g/day	ND	Glomerular TMA	IgA, MES	EDDs, SEn and focally MES	Persistent proteinuria
5	70/M	2 w	6.0 g/gCr	ND	Glomerular TMA	No	ND	Proteinuria decreased
9	59/M	15 mo	6.9 g/day	−	Glomerular TMA	IgA, MES and capillary	EDDs, sparsely SEn	Proteinuria decreased
10	ND/ND	ND	Nephrotic	ND	Cryo-GN	ND	ND	ND
11	ND/F	ND	Nephrotic	ND	Collapsing GP	ND	ND	ND
12	69/F	3 mo	3.6 g/day	−	Glomerular TMA + collapsing GP	IgA, capillary	SEn widening without EDDs	Proteinuria decreased
13	71/M	1 mo	9.6 g/day	ND	Focal PGN	C3 and IgM, MES and capillary	EDDs, sparsely SEn	Proteinuria decreased
14	ND/M	3 mo	3.4 g/gCr	ND	Glomerular TMA	ND	ND	ND
14	ND/ND	1 mo	3.2 g/gCr	ND	Glomerular TMA	ND	ND	ND
Present case	64/M	14 mo	3.5 g/day	+	MES PGN + TMA	IgA, MES and capillary	EDDs, MES and SEn	Proteinuria resolved

Foot notes of Table 3.

Onset means the duration form the start of bevacizumab to the appearance of urinary abnormalities.

*M*, male, *F*, female, *LM*, light microscopy, *Cr*, serum creatinine, *IgA*, immunoglobulin A, *IgM*, immunoglobulin M, *C3*, serum complement component 3, *IF*, immunofluorescence, *EM*, electron microscopy, *TMA*, thrombotic microangiopathy, *MES*, mesangial, *SEn*, subendothelial, *Cryo-GN*, cryoglobulinemic glomerulonephritis, *GP*, glomerulopathy, *PGN*, proliferative glomerulonephritis, *FPE*, foot process effacement, *EDDs*, electron dense deposits, *ND*, not documented, *mo*, month(s), *w*, week(s).

The mechanisms by which bevacizumab caused IgA nephropathy are unknown. It is easy to eliminate the possibility that IgA immune deposits comprised the bevacizumab–VEGF immune complex, because bevacizumab is a recombinant humanized murine antibody of the IgG1 subclass [12], and IgG deposition in this case was very mild. One possibility is that circulating IgA immune complexes accumulated in the mesangium and subendothelial areas through capillary walls damaged by bevacizumab. However, the accumulation of IgA immune complexes may depend on factors unique to each patient, such as the amount of circulating IgA immune complexes, the presence or absence of chronic mucosal inflammation, or environmental factors, because not all patients with bevacizumab-induced TMA exhibited glomerular IgA deposition. In our case, serum IgA gradually decreased after bevacizumab cessation (512, 295, 277, and 270 mg/dl at 1, 12, 15, and 25 months after cessation, respectively). To date, no reports describe serum IgA in bevacizumab-induced renal diseases. Although the effect of bevacizumab on the immune system is unclear, further analyses of bevacizumab and serum IgA are warranted, as bevacizumab reportedly affects the permeability of gastroduodenal and intestinal mucosa [17], which is known to produce mucosal IgA [18] and to be closely associated with IgA nephropathy development [19].

In conclusion, this is the first case report that confirmed histologically the development and resolution of IgA nephropathy during and after bevacizumab therapy. Although anti-VEGF effect on the permeability of gastroduodenal and intestinal mucosa might be related to the IgA nephropathy development, further studies and accumulation of cases are warranted to elucidate the exact mechanism.

## Consent

Written informed consent was obtained from the patient for the publication of this case report and accompanying images. A copy of the written consent is available for review by the Editor–in-Chief of this journal.

## Abbreviations

CEA: Carcinoembryonic antigen; C: Complement component; EDDs: Electron dense deposits; GN: Glomerulonephritis; Ig: Immunoglobulin; IF: Immunofluorescence; HPF: High-power field; PAS: Periodic acid-Schiff; TMA: Thrombotic microangiopathy; VEGF: Vascular endothelial growth factor.

## Competing interests

The authors declare that they have no competing interests.

## Authors’ contributions

MY performed the renal biopsy and follow-up of the patient. MY, IN, and JS were involved in drafting the manuscript, and JS carried out final preparation of the manuscript. TS, HS, and SA participated in the discussion of histomorphological and electron microscopy diagnosis. All authors read and approved the final manuscript.
